# N uptake, assimilation and isotopic fractioning control δ ^15^N dynamics in plant DNA: A heavy labelling experiment on *Brassica napus* L.

**DOI:** 10.1371/journal.pone.0247842

**Published:** 2021-03-11

**Authors:** Alessandro Foscari, Giulia Leonarduzzi, Guido Incerti

**Affiliations:** 1 University of Trieste, Trieste, Italy; 2 Department of Agricultural, Food, Environmental and Animal Sciences, University of Udine, Udine, Italy; Southern Cross University, AUSTRALIA

## Abstract

In last decades, a large body of evidence clarified nitrogen isotope composition (δ^15^N) patterns in plant leaves, roots and metabolites, showing isotopic fractionation along N uptake and assimilation pathways, in relation to N source and use efficiency, also suggesting ^15^N depletion in plant DNA. Here we present a manipulative experiment on *Brassica napus* var. *oleracea*, where we monitored δ ^15^N of purified, lyophilized DNA and source leaf and root materials, over a 60-days growth period starting at d 60 after germination, in plants initially supplied with a heavy labelled (δ ^15^N_Air-N2_ = 2100 mUr) ammonium nitrate solution covering nutrient requirements for the whole observation period (470 mg N per plant) and controlling for the labelled N species (ṄH_4_, ṄO_3_ and both). Dynamics of Isotopic Ratio Mass Spectrometry (IRMS) data for the three treatments showed that: (1) leaf and root δ ^15^N dynamics strictly depend on the labelled chemical species, with ṄH_4_, ṄO_3_ and ṄH_4_ṄO_3_ plants initially showing higher, lower and intermediate values, respectively, then converging due to the progressive NH_4_^+^ depletion from the nutrient solution; (2) in ṄH_4_ṄO_3_, where δ^15^N was not affected by the labelled chemical species, we did not observe isotopic fractionation associated to inorganic N uptake; (3) δ^15^N values in roots compared to leaves did not fully support patterns predicted by differences in assimilation rates of NH_4_^+^ and NO_3_^-^; (4) DNA is depleted in ^15^N compared to the total N pools of roots and leaves, likely due to enzymatic discrimination during purine biosynthesis. In conclusion, while our experimental setup did not allow to assess the fractionation coefficient (ε) associated to DNA bases biosynthesis, this is the first study specifically reporting on dynamics of specific plant molecular pools such as nucleic acids over a long observation period with a heavy labelling technique.

## Introduction

In last decades a large body of evidence clarified the main sources of variation in nitrogen isotope composition (δ ^15^N) at intra-plant level (review in [1]), showing that isotopic fractionation occurs in relation to the chemical species, content and bioavailability of inorganic and organic N in the substrate, with known biochemical mechanisms [2].

Plant isotope composition firstly relates to δ ^15^N and relative fractions in the substrate of different N sources, such as NH_4_^+^, NO_3_^-^, organic N, or N_2_ in the case of species symbiotic with diazotrophic prokaryotes [3]. Plant δ ^15^N also varies compared to that of soil N due to different uptake mechanisms, assimilation pathways, and rates of N recycling, which can all discriminate against the heavy isotope [1]. As an example, NO_3_^-^ uptake is mediated by either constitutive carrier system with high-substrate affinity or non-saturable transport mechanisms with low-substrate affinity, which act at low (0–500 μM) and high (> 500 μM) NO_3_^-^ concentrations in the substrate, respectively. Both transport systems produce isotopic fractionation [4–6], although plant-to-soil δ ^15^N variation is larger when NO_3_^-^ is the primary N source, and smaller when NH_4_^+^ is used [1]. On the other hand, species- and cultivar-specific effects can outcompete those of the N chemical species [5, 7], which led to measuring foliar δ ^15^N to understand the physiological mechanisms underlying N use differences among co-occurring species [1].

Considering discrimination against ^15^N during inorganic N assimilation [3], several previous studies focused on the enzymatic fractionation by nitrate reductase and glutamine synthetase [8–12] and its effects on intra-plant δ ^15^N variation. Nitrate is assimilated both in roots and leaves, where the content of assimilation enzymes and the rate of assimilation can affect the resulting δ ^15^N [13]. However, the NO_3_^-^ available for assimilation in leaves is enriched relative to root NO_3_^-^ because it originates from a pool that has already been exposed to fractionation during root assimilation, leading to higher δ ^15^N of leaves compared to roots [1]. However, it has been recently reported that NO_3_^-^ can be enriched in ^15^N in roots compared to leaves, due to nitrate circulation and compartmentalization, in particular by phloematic backflow from the leaves [14]. Differently, NH_4_^+^ is immediately assimilated in the root, therefore root vs. leaf δ ^15^N differences are less affected by NH_4_^+^ assimilation, as organic nitrogen in shoots and roots is the product of a single assimilation event [1].

Further contributions to intra-plant δ ^15^N variation rely on isotopic fractionation during xylematic [15] and re-allocation [16] flows, as well as N depletion by NH_3_ and NO_2_ volatilization, although the latter process, being limited to the leaf senescence stage, likely bears negligible effects [12]. Finally, the role of plant symbionts such as mycorrhizae and N-fixing rhizosphere bacteria were investigated in both field and controlled conditions [17], showing interesting dynamics [18] but limited effects, in relation to their negligible mass compared to that of the plant [19].

More recently, isotopic fractionation has been investigated along specific metabolic pathways by IRMS analysis after purification of different leaf metabolites, including amino acids, nucleic acids and chlorophylls [20] or by compound-specific stable isotope analysis (CSIA), where IRMS is coupled with GC-MS or LC-MS interface to separate different metabolites before isotopic analysis [21–23]. Differences of δ ^15^N among different molecular N pools depend on isotopic discrimination by most enzymes of primary N metabolism [e.g. Glu synthase, transaminases, Asn synthetase, etc., 10]. Accordingly, Gauthier et al. observed by CSIA a different δ ^15^N in different N molecular pools in *Brassica napus* leaves, corresponding to a predominant effect of enzymatic discrimination in amino acid metabolic pathways, compared to that associated to the inorganic N source [20]. Moreover, the N pool of leaf DNA, purified by standard methods, lyophilized and isotopically analyzed by EA-IRMS, was isotopically depleted compared to amino acids, consistent to discrimination associated with the synthesis of bases [20]. Several enzymes involved in pyrimidine synthesis discriminate the heavy isotope, such as carbamoyl phosphate synthetase [24], aspartate carbamoyltransferase [25], dihydroorotase [26], orotate phosphoribosyltransferase [27]. In the case of purine synthesis, amino acids such as Glu, Gln, Asp, and Gly are the N sources, but isotope effects along these metabolic ways are not yet fully clarified. Consequently, the δ ^15^N of leaf DNA is expected to be lower compared to other leaf N pools [20]. Accordingly, lower δ ^15^N is expected in plant DNA compared to the source material from which it is purified, whose δ ^15^N value results from the average of different molecular pools, weighted by their relative mass fraction [28]. However, changes of δ ^15^N in plant materials during plant development could affect the expected pattern, as related to possible decoupling in time of DNA biosynthesis and inorganic N uptake and assimilation dynamics, and hence of their fractionation effects.

Therefore, in this study we set up a manipulative experiment in controlled conditions on *Brassica napus* var. *oleracea*, monitoring δ ^15^N of purified DNA and source leaf and root materials, over a 60-days growth period starting at d 60 after germination, in plants initially supplied with a heavy labelled ammonium nitrate solution and controlling for the labelled N species (either NO_3_^-^, NH_4_^+^ or both). We assumed that the magnitude of isotope effects is small enough that they generally do not perturb plant growth dynamics when compared to the unlabelled control [29]. Our specific hypotheses and expected outcomes were that: (1) leaf and root δ ^15^N dynamics strictly depend on the labelled chemical species, as related to a limiting effect of NH_4_^+^ concentration on the uptake of NO_3_^-^ [8]. Accordingly, plants supplied with either labelled NH_4_^+^, labelled NO_3_^-^ or both labelled species (thereafter referred to as ṄH_4_, ṄO_3_ and ṄH_4_ṄO_3_, respectively) should initially show higher, lower and intermediate values, respectively. Then, the progressive NH_4_^+^ depletion from the nutrient solution should correspond to an increase of NO_3_^-^ uptake rate, with ṄH_4_ and ṄO_3_ plant materials showing progressively decreasing and increasing δ ^15^N, respectively. (2) In ṄH_4_ṄO_3_ plants, where δ ^15^N is not affected by the labelled N chemical species, we tested the occurrence of isotopic fractionation associated to inorganic N uptake [4, 6, 30], expecting an increase of δ ^15^N over time due to a progressive ^15^N enrichment in the N pool residual in the pot solution. (3) Differences in assimilation rates in roots compared to leaves should produce, at a given observation stage, higher δ ^15^N values in ṄH_4_ roots compared to ṄH_4_ leaves and in ṄO_3_ leaves compared to ṄO_3_ roots, with ṄH_4_ṄO_3_ materials showing intermediate values; (4) Consistent to expectations [20], DNA is depleted in ^15^N compared to the other molecular N pools, and thus to the source plant material, due to enzymatic discrimination during purine biosynthesis.

## Materials and methods

**Fig 1:**
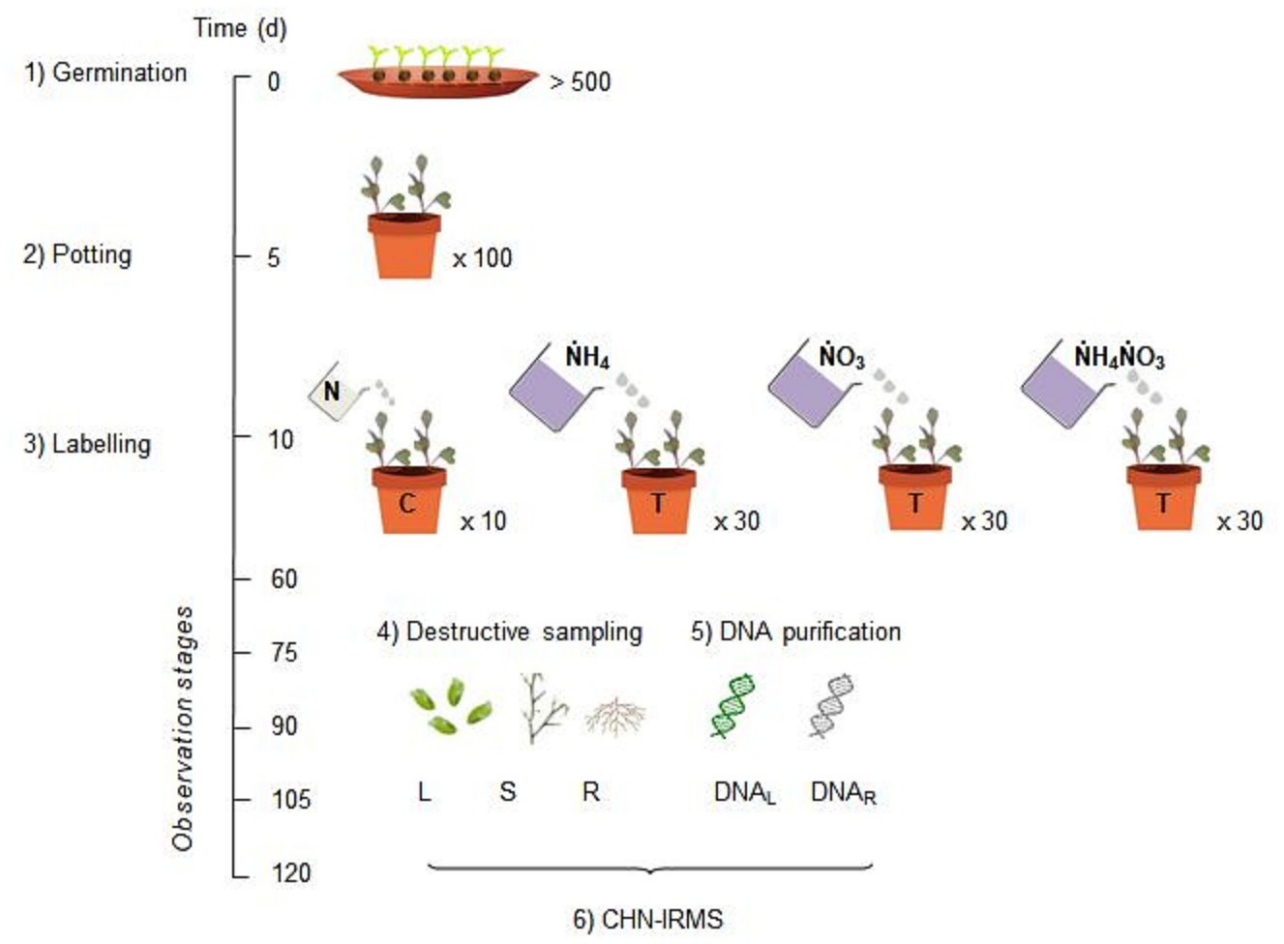
Illustration of the experimental design. Six manipulative steps are shown along the experiment timeline: 1) seeds germination at d 0; 2) potting at d 5; 3) labelling with ammonium nitrate solution, including 3 treatments (T, each on 30 replicated pots) with the same isotopic ratio, i.e. δ^15^N_Air-N2_ = 2100 mUr, but differing by the labelled chemical species (ṄH_4_, ṄO_3_ or ṄH_4_ṄO_3_), plus the untreated control (C, 10 replicates) administered with the same dose of unlabelled ammonium nitrate (N); 4) destructive sampling (6 replicates per treatment at each of 5 observation stages from d 60 to d 120) and separation of leaf, stem and root materials (L, S and R, respectively); 5) DNA extraction and purification from leaf ad root materials; 6) CHN-IRMS analysis of dry aliquots of plant materials and corresponding DNA samples. See methods for further details.

Our experimental design is summarized in Fig 1, including seed germination and potting, isotopic labelling, periodic destructive sampling, plant DNA extraction and purification and CHN-IRMS analysis.

### Plant material, sowing and potting

Commercial seeds of *Brassica napus* var. *oleifera*, cultivar Gordon (KWS SAAT SE & Co. KGaA, Germany), were imbibed with Milli-RO water for 24 h into in 50 mL lab grade tubes, then transferred to plastic saucers filled with dried, quartz sand substrate (GESTECO Spa, Italy; physical-chemical features in S1 Table) and kept in a growing room in controlled optimal conditions (T = 22/20 °C, photoperiod 12 h, RH = 50%, PAR 600 μmol photons m^-2^ s^-1^). Five days after germination (i.e. d 5), seedlings were transplanted in pots (2 seedlings per pot) previously filled with 1.3 kg of substrate. Each pot had draining holes allowing drainage and preventing hypoxia, and was placed on a saucer to prevent nutrient loss. One-hundred pots were considered, corresponding to 30 replicates for each of 3 labelling treatments, plus 10 unlabelled controls.

### Labelling and nutrient solution

Three different N labelling treatments with different labelled chemical species (either ammonium, nitrate or both) were considered, all with equal isotopic ratio (δ ^15^N_Air-N2_ = 2100 mUr). Such extremely high value was used in order to ensure the detectability of ^15^N depletion in leaf and root DNA along the observation period, in absence of previous quantitative evidence on the possible enzymatic discrimination coefficient [20]. Ammonium nitrate solutions for labelling were prepared by mixing a water solution of commercial NH_4_NO_3_ (Sigma-Aldrich, USA, δ ^15^N_Air-N2_ = 0.7 mUr) with that of each labelled salt (Cambridge Isotope Labs, 98% of labelled atoms) in the opportune mixing ratio, following the equations reported in [28].

At d 10, each of 30 potted seedlings for each labelling treatment was supplied with 50 mL of a 0.336 M ammonium nitrate solution (i.e. 470 mg of N per pot), corresponding to N requirements for a 180-days growing period, estimated according to previous reports on dynamics of leaf and root growth, N content and uptake efficiency [31–34]. In this way, it was possible to assess the labelling dynamics of plant materials since an initial starting point of known δ ^15^N of the nutrient solution.

Other macro- and micro-nutrient were also supplied at d 10, proportionally to N according to the following modified Hoagland solution (280 mL per pot): 10 mM MgSO_4_, 1 mM Fe(Na)-EDTA, 20 μM KCl, 0.5 mM H_3_BO_3_, 40 μM MnSO_4_, 40 μM ZnSO_4_, 2 μM CuSO_4_, 2 μM (NH_4_)_6_Mo_7_O_24_, 4 mM CaSO_4_, 20 mM K_2_HPO_4_, 60 mM K_2_SO_4_. Before supply, the nutrient solution was buffered with MES (2-(N-morpholino)ethanesulfonic acid, 40 mM) and pH was corrected at 6.0±0.1 with HCl 4M. After d 10, no further nutrient was administered to the pots, with the exception of CaSO_4_, since its low solubility made impossible to fulfill plant requirements with the initial dose at d 10. Therefore, CaSO_4_ was supplied (at 4 mM per pot) over the growing period while watering (see next section), for a total of 1.524 g/plant.

It is worth noting that, all together, the ion strength of the nutrient solutions was extremely high (over 260 mM), especially immediately after the nutrient supply at d 10. While this posed issues related to osmotic stress, preliminary tests had showed that *B*. *napus* seedlings were capable to survive such stressing conditions. Therefore, although possibly misrepresenting physiological conditions during plant growth in nature/field, our approach, with most nutrient supply at the beginning of the growing period was the only choice allowing to monitor the labelling dynamics of plant tissues and DNA at medium term (120 d).

### Plant cultivation

In the growing room, pots were randomly (i.e. independent of the labelling treatment) placed onto five trolleys. Trolley within the room and pots within each trolley were daily and weekly moved, respectively, to keep homogeneous exposure condition among replicates. Water loss by evapotranspiration was reintegrated by watering the pots every two days with milli-RO water. At d 15, pot thinning by uprooting the less developed seedling allowed to maintain a replicate for each treatment while avoiding possible confounding effects of within-pot intraspecific competition.

### Destructive sampling

Starting at d 60 and every 15 days until d 120, 6 pots per treatment and 2 control pots were randomly selected and plants uprooted. Roots were gently washed with deionized water in order to remove sand particles and residues of nutrient solution. Afterwards, roots, stems (petioles) and leaf materials of each plant were separated.

Fresh plant materials (i.e. leaves, stems and roots) of each sampled pot were separately weighted. Afterwards, aliquots (i.e. 15 mm-diameter discs, 2 cm-long segments and a portion of the tip for each leaf blade, stem and root, respectively) were collected, fresh-weighted, dried in stove (24 hrs. at 60°C), dry-weighted, pulverized (TissueLyser II, Qiagen, Hilden, Germany) and kept in sterile plastic tubes for CHN-IRMS analyses. Residual, fresh plant materials (5 g each) were separately ground (Mill A11 basic, IKA, Saint Louis, Missouri, USA) in liquid nitrogen (T = -196 °C), placed in sterile 50 mL Falcon tubes and stored in freezer at -80 °C for subsequent DNA extraction.

Since fresh plant materials are required for DNA extraction, the shoot: root ratio of each plant was determined as the ratio between the total dry leaf and stem biomass and the total dry root biomass of each sampled plant, with dry weights estimated based on the fresh weight: dry weight ratio of the aliquots.

### DNA extraction, purification and quantitation

DNA extraction from fresh leaves and roots of each plant followed a modified version of the Doyle & Doyle protocol [35], as follows: a lysis buffer was prepared mixing 20 mL of CTAB (2.5%), a spatula tip of PVP-40, 2 μl of Proteinase K (20 μg/μl) and 200 μl of β-mercaptoetanol (0.1%). The buffer solution was kept in agitation in a thermostatic bath at 65 °C (pbi, Braski, Bergamo, Italy), until PVP complete dissolution. For each source plant material, 20 mL of the buffer solution were added to the Falcon tube and the mixture was incubated at 65 °C for 30 min. and successively cooled in ice for 10 min. DNA purification was performed adding 20 mL of a mixture of chloroform: isoamyl alcohol (24:1) and gently shacking for 10 min. to homogenize. Falcon tubes containing the mixture were centrifuged at 6800 rpm at 4°C for 30 min, then gently pipetting out the aqueous supernatant fraction. Sodium acetate (1/10 starting volume, 3M), NaCl (3/10 starting volume, 4M) and pure Isopropyl alcohol (2/3 final volume) were added to the collected supernatant. The solution was incubated at -20°C for 30 min. and successively centrifuged as described above. As final step, after removing the supernatant and twice washing the pellet with 2 mL ethanol (80%), the pellet was dried in a stove (10–15 min. at 37 °C) and re-suspended into an Eppendorf tube filled with 1.7 mL of sterile water for quantitation.

The concentration of extracted DNA in the resuspension medium was assessed by fluorimeter Qubit 3.0 (Life Technology, Carlsbad, California, USA). Finally, aliquots of 1.5 mL of each DNA sample were collected, frozen, lyophilized (55–4 Coolsafe, Scanvac, Allerød, Denmark) for 24 h (0.050 mbar, T = -57 °C) and kept in sterile plastic tubes at -20°C or subsequent CHN-IRMS analyses.

### CHN-IRMS analysis

Dry, pulverized root and leaf samples as well as lyophilized DNA samples purified from the same materials were weighted at 2 ± 0.5 mg in cylindrical tin capsules (diameter 5 mm, height 9 mm) (Säntis Analytical AG, Teufen, Switzerland) in duplicates. A total of 800 replicated samples (100 plants x 2 plant materials x 2 technical replicates x 2 N pools, i.e. total N and DNA) were processed by elemental analysis/isotope ratio mass spectrometry (EA/IRMS), using a Vario Micro Cube (Elementar GmbH, Langenselbold, Germany) elemental analyzer connected online in continuous flow mode to an IsoPrime 100 (Elementar UK Ltd, Cheadle Hulme, UK) isotope ratio mass spectrometer, using helium (He) as a carrier gas.

Flash combustion of all samples was conducted at 950 °C with a pulse of O_2_ (30 ml/min for 70’) into the He carrier gas in a quartz combustion column prepared following the manufacturer instructions. From bottom to top, the column was filled with: quartz wool (two layers, each of height 2.5 mm, separated by a 18 mm- thick layer of quartz chips), silver wool (25 mm), quartz wool (5 mm), CuO (65 mm), corundum balls (3 mm), an ash-finger tube with Al_2_O_3_ bottom, and a sheath tube. The combustion gas products (CO_2_, N_2_, NO_x_ and H_2_O) were passed through a reduction column at 500 °C to reduce the non-stoichiometric nitrous products to N_2_ and to remove excess oxygen from the gas stream. The reduction column was prepared following the manufacturer instruction and filled with quartz wool at the bottom (5 mm height), elemental copper (295 mm), silver wool (20 mm). The plug of the reduction column was also filled with silver wool, to bind volatile halogen compounds contained in the combustion gas products. Reduced gases were then dried by passing through a 10 cm glass column filled with anhydrous SICAPENT^®^ (Merk, Darmstadt, Germany), then passing into desorption columns to absorb the measurable components of the analysis gas mixture and then release each of them by controlling the desorption temperatures. Once released, the gases sequentially passed through a Thermal Conductivity Detector (TCD) and were vented out to the Isoprime diluter (Elementar UK Ltd, Cheadle Hulme, UK) for diluting CO_2_ flow in the carrier helium flow at a rate of 100 ml/min before entering in the mass spectrometer. In parallel to this sample line, a second helium line is connected to the source of the mass spectrometer to carry the two calibration gases (CO_2_, N_2_). Isotopic measurements and data processing were performed with the software IonVantage (Elementar UK Ltd, Cheadle Hulme, UK).

The nitrogen stable isotope composition in a given sample can be reported in the delta (δ) notation as variations of the molar ratio (R) of the heavy (^15^N) to light isotope (^14^N) in the sample relative to molecular nitrogen in air (Air-N_2_) as international standard [36]. Accordingly, we used the following notation:

$${\delta^{15}N}_{Air-N_{2}}=\frac{{R{(}^{15}N{/}^{14}N)}_{sample}}{{R{(}^{15}N{/}^{14}N)}_{Air-N_{2}}}-1$$

The unit commonly used to express the delta value is permil (‰). However, the use of ‰ is debated as in conformity with the International System of Units (SI) and according to the guidelines and recommendations of the International Union of Pure and Applied Chemistry (IUPAC)—Commission on Isotopic Abundances and Atomic Weights [37, 38], the unit of the delta values is the “urey” (symbol Ur). Therefore, we presented values of nitrogen isotopic composition with the unit notation mUr. However, as 1 mUr equals 1 ‰, for the sake of compliance with previous studies, we also presented isotopic composition values with the double unit notation of “mUr or ‰” limited to figures and tables, as often reported in the literature [e.g. 39, 40].

Analytical results for nitrogen isotopic composition were calibrated using sulphanilamide (Elementar GmbH, Langenselbold, Germany, N = 16.26%, C = 41.81%, 7 samples per batch) as a reference material. Analytical results for nitrogen isotopic composition were linearly corrected using the following international reference materials (International Atomic Energy Agency, Wien, Austria): IAEA-N1 (ammonium sulphate, δ^15^N_Air-N2_ = 0.4 mUr, 4 samples per batch): IAEA-305A (ammonium sulphate, δ^15^N_Air-N2_ = 39.8 mUr, 4 samples per batch), IAEA-310A (urea, δ^15^N_Air-N2_ = 47.2 mUr, 2 samples per batch), USGS 26 (ammonium sulphate, δ^15^N_Air-N2_ = 53.7 mUr, 2 samples per batch), IAEA-310B (urea, δ^15^N_Air-N2_ = 244.6 mUr, 4 samples per batch), IAEA-305B (ammonium sulphate, δ^15^N_Air-N2_ = 375.3 mUr, 4 samples per batch). Each analytic batch (120 positions) included 90 samples, 27 reference materials, 2 blanks consisting in empty tin capsules and 1 empty position. To avoid possible concerns of memory effects in the analytic results due to isotopically enriched samples, blanks were measured at the beginning of the batch and samples were sequentially placed in each batch according to the expected isotopic enrichment for different types of samples, thus minimizing the enrichment gaps between each sample type. Furthermore, duplicates of the same source sample were always placed in different batches to increase accuracy. The repeatability and intermediate precision of the EA/IRMS were determined by the standard deviation of separately replicated analyses and were better than 0.1 mUr.

### Data analysis

All statistical analyses were performed using the software Statistica 7.0 (StatSoft inc., Tulsa, Oklahoma, USA). Generalized linear models (GLMs) were fitted for leaf, root and stem biomass and N content, considering main and interactive effects of the labelling treatment (three levels: ṄH_4_ṄO_3_, ṄH_4_, ṄO_3_), plant material (three levels: leaves, stems and roots) and plant age, the latter included in the model as a continuous covariate. For each plant material and age, significant differences between treatments and control plants were tested using Tuckey’s HSD post-hoc test.

In order to compare δ ^15^N of purified DNA to that of the source plant materials, we calculated a Normalized Difference Index (NDI) for each replicate and treatment combination (i.e. labelling treatment, plant material and age), as follows:

$${NDI}_{i,j,k,n}=\frac{\delta \;{}^{15}N{\left(DNA\right)}_{i,j,k,n}-\delta \;{}^{15}N{\left(Source\;material\right)}_{i,j,k,n}}{\delta \;{}^{15}N{\left(DNA\right)}_{i,j,k,n}+\delta \;{}^{15}N{\left(Source\;material\right)}_{i,j,k,n}}$$

where *i*, *j*, *k* and *n* indicates the labelling treatment, the type of source plant material (either leaf or root), plant age and the experimental replicate (individual plant), respectively. As such, NDI values range between -1 and 1, corresponding to unlabelled (δ ^15^N_Air-N2_ = 0 mUr) DNA and source plant material, respectively, while NDI = 0 corresponds to equal δ ^15^N values of DNA and total N pool of the source plant material.

GLMs were fitted for δ ^15^N of DNA and source plant materials, and for NDI as well, considering main and interactive effects of the labelling treatment (three levels: ṄH_4_ṄO_3_, ṄH_4_, ṄO_3_), plant material (two levels: leaves, stems and roots) and plant age, the latter included in the model as a continuous covariate. For all GLMs, pair-wise significant differences between treatment combinations were evaluated by Tuckey’s post-hoc HSD test at α = 0.05. Limited to NDI data, mean values of different experimental groups (i.e. unique combinations of plant material, age and labelling treatment) were also tested for significant difference from the reference zero value by one-sample *t* test at α = 0.01, thus reducing the conventional level of statistical significance of 0.05 in order to control for multiple comparisons. As such, significant negative and positive NDI mean values were interpreted as indicating ^15^N depletion and enrichment in DNA, respectively, compared to the total N pool of the source plant materials.

## Results

### δ ^15^N dynamics in labelled plant leaves and roots

**Fig 2:**
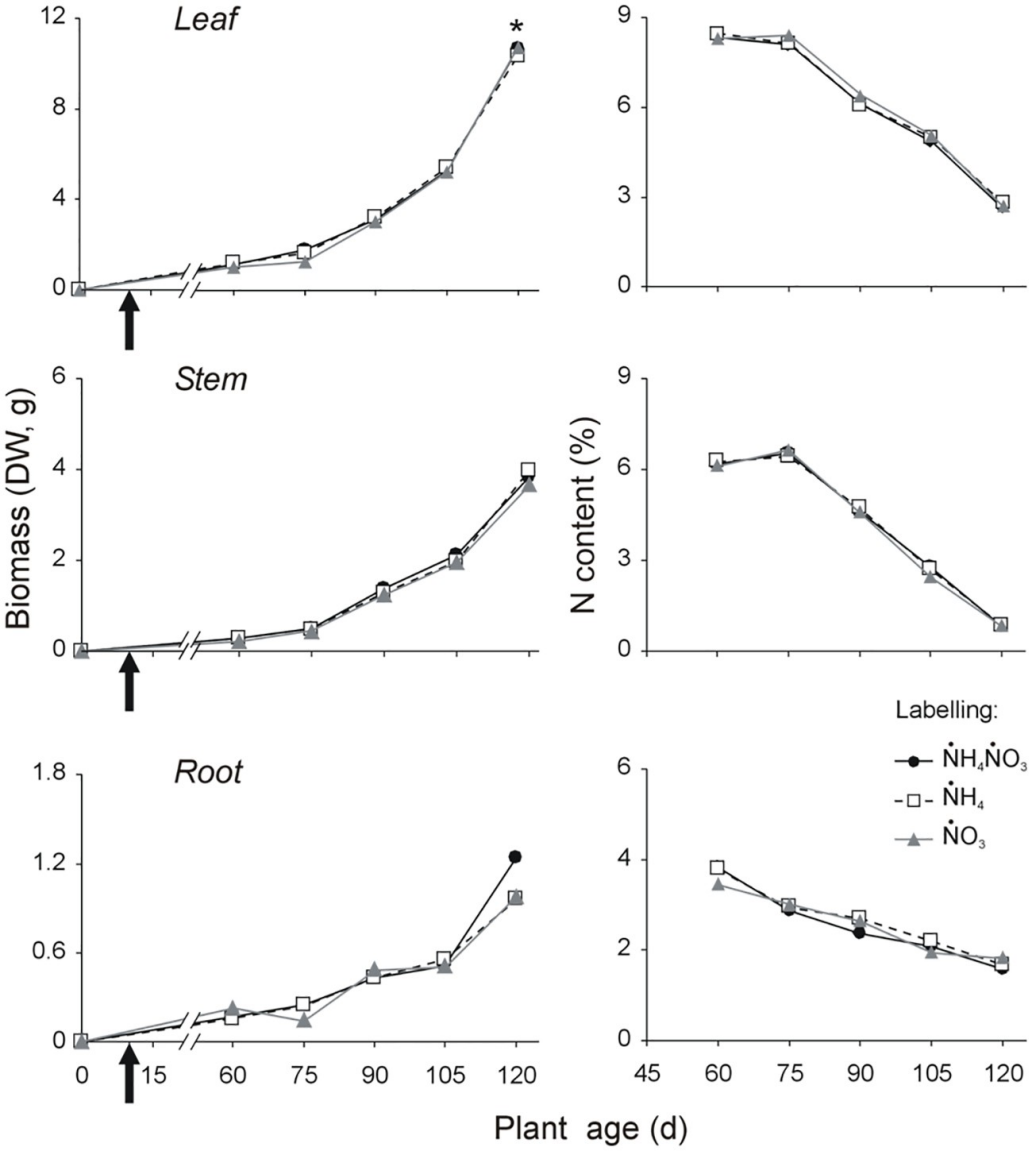
Dynamics of dry biomass and percent N content in *B*. *napus* leaves, stems and roots of plants grown for 120 days in controlled conditions and fertilized with ammonium nitrate according to different N isotopic labelling treatments differing by the labelled chemical species (ṄH_4_, ṄO_3_, or both) but with the same isotopic ratio (δ^15^N_Air-N2_ = 2100 mUr). Arrows on the left panels indicate the labelling administration date at plant age of 10 d. Data refer to mean of 6 plants for each treatment combination. Deviation bars are omitted to improve readability. Statistical support in S2, S3 and S4 Tables.

Labelling treatments did not affect plant growth (Fig 2, S3 Table), percent N content (Fig 2, S4 Table) and biomass allocation (S1 Fig, S6 and S7 Tables). Leaf, stem and root biomasses, as well as their percent N content, were not significantly different among labelling treatments and between them and the unlabelled control plants (S5 Table), with the exception of leaf biomass at 120 d, which was significantly lower in plants of the three labelling treatments (Fig 2 and S5 Table, Tuckey’s HSD test: p-values < 0.0001 in all three pairwise treatment vs. control comparisons), possibly due to the interplay of individual variability and low sample size of the control plants. Finally, labelling treatments did not affect shoot: root ratio (S1 Fig, S6 and S7 Tables).

**Fig 3:**
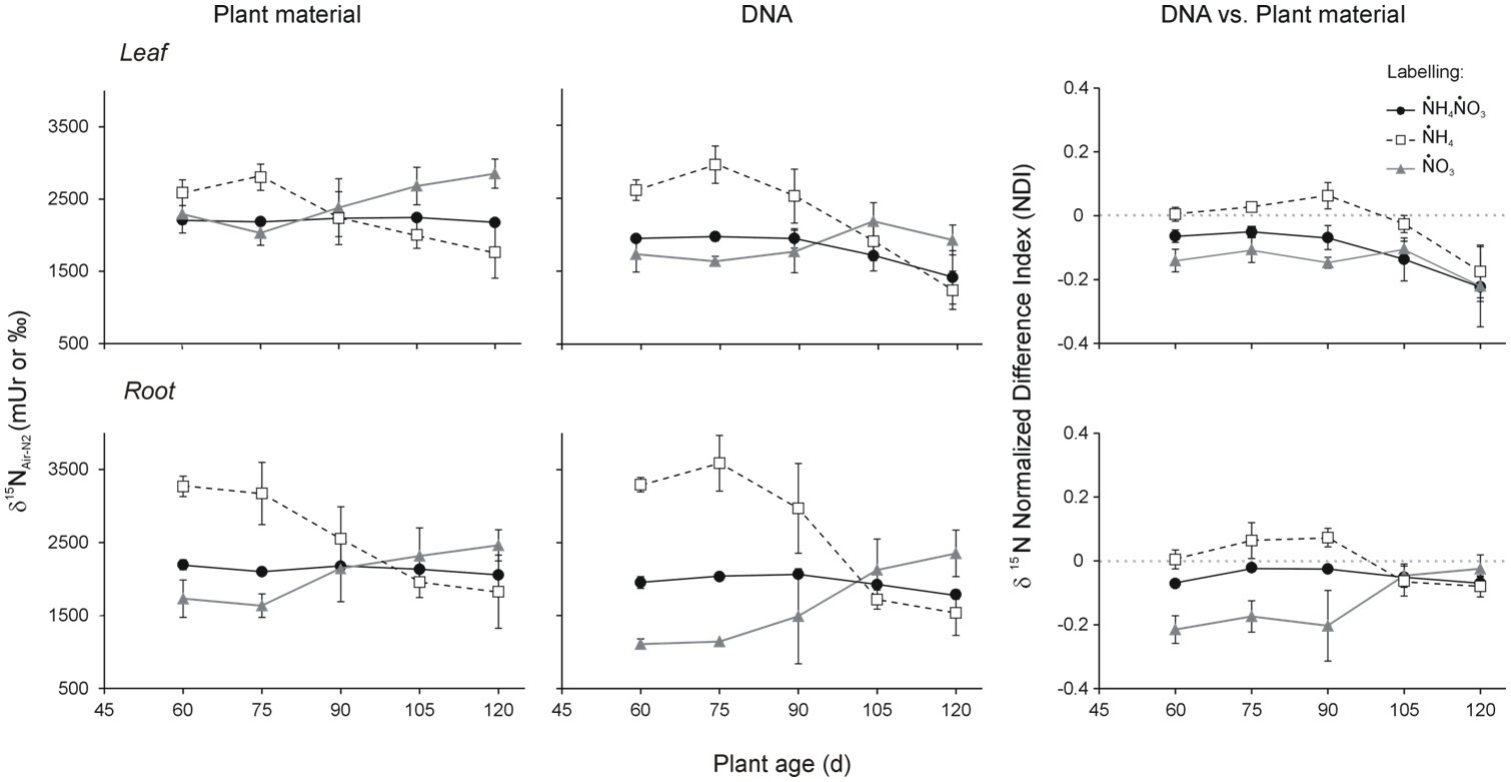
Dynamics of N isotopic composition in *B*. *napus* leaves and roots (left) and DNA samples extracted therefrom (center) across the labelling treatments. Right panels show the corresponding δ ^15^N Normalized Difference Index (NDI) dynamics. Data refer to mean of 6 plants for each treatment combination. Statistical support in Tables 1 and 2, and S8, S9 and S10 Tables.

δ ^15^N of leaves and roots largely varied with plant age and among labelling treatments (Fig 3), as indicated by the significant interaction term in the GLM model (Table 1).

**Table 1:** Results of GLM for δ ^15^N of plant materials and DNA extracted thereof.

Effect	DoF	SS	MS	F	*p*
***δ*** ^***15***^***N of plant materials***					
Labelling treatment (L)	2	17690704	8845352	124.538	< 0.0001
Plant material (M)	1	132441	132441	1.865	0.1739
Plant age (A)	1	837738	837738	11.795	0.0007
L × M	2	1433748	716874	10.093	< 0.0001
L × A	2	16619336	8309668	116.995	< 0.0001
M × A	1	226831	226831	3.194	0.0758
L × M × A	2	649352	324676	4.571	0.0117
Error	168	11932294	71026		
***δ*** ^***15***^***N of DNA***					
Labelling treatment (L)	2	36223363	18111682	166.326	< 0.0001
Plant material (M)	1	198035	198035	1.819	0.1793
Plant age (A)	1	4105552	4105552	37.703	< 0.0001
L × M	2	3711058	1855529	17.040	< 0.0001
L × A	2	27770355	13885178	127.513	< 0.0001
M × A	1	382620	382620	3.514	0.0626
L × M × A	2	2607457	1303728	11.973	< 0.0001
Error	168	18293926	108892		

GLMs include main and interactive effects of labelling treatment (L, three levels: ṄH_4_ṄO_3_, ṄH_4_ and ṄO_3_), plant material (M, two levels: leaf and root) and age (A, continuous covariate).

In leaves, initially (60 d) δ ^15^N did not show significantly different values among ṄH_4_ṄO_3_, ṄH_4_ and ṄO_3_ plants. At the second observation stage (75 d), a slight increase in ṄH_4_ and a decrease in ṄO_3_ plants were detected (Fig 3), but still not statistically significant compared to the previous stage in both treatments (S8 Table). Since 75 d, δ ^15^N dynamics differed among the three treatments (Fig 3), with ṄH_4_ leaves showing a progressive decrease, down to -37.0% from 75 d to 120 d, while ṄO_3_ leaves showed the opposite pattern, increasing by 40.5% in the same time frame (S8 Table). Differently, ṄH_4_ṄO_3_ leaves did not show significant δ ^15^N changes along the observation period (S8 Table). Interestingly, after 120 d observation δ ^15^N in ṄO_3_ leaves significantly exceeded that of ṄH_4_ṄO_3_ ones by 31.1% and that of ṄH_4_ leaves by 61.5%, while the difference between the mean values of the two latter treatments was not statistically significant, with ṄH_4_ leaves showing high within-treatment variability (S8 Table).

δ ^15^N dynamics in roots were qualitatively similar to those observed for leaves (Fig 3). However, significant 2^nd^ order interactive effects of the types of plant material and labelling treatment, and of the 3^rd^ order interaction with plant age as well (Table 1), indicated that δ ^15^N values in plant roots, within each labelling treatment, followed quantitatively different dynamics compared to leaves. In particular, significant differences among ṄH_4_ṄO_3_, ṄO_3_ and ṄH_4_ roots were observed since the beginning of the observation period (S8 Table), with the latter treatment producing δ ^15^N values exceeding those of ṄH_4_ṄO_3_ and ṄO_3_ roots by 49.0% and 88.8%, respectively. Such trend still held at 75 d, with corresponding percent differences of 51.1% and 94.0%, respectively.

Thereafter, from 90 d to 120 d, root δ ^15^N dynamics were apparently similar to those observed in leaves (Fig 3), although ṄO_3_ roots did neither show statistically significant age-dependent variations, nor significant differences compared to the other treatments within each observation stage (S8 Table). The only exception to such pattern was the significantly higher δ ^15^N values at 120 d in ṄO_3_ vs. ṄH_4_ roots (+34.8%), resulting from the decreasing age-dependent trend observed in this latter treatment (-42.4% from 75 d to 120 d). As observed in leaves, ṄH_4_ṄO_3_ roots did not show significant δ ^15^N changes along the observation period (S8 Table).

Finally, δ ^15^N differences between root and leaf materials within each labelling treatment at each observation stage were not statistically significant with the exception of ṄH_4_ and ṄO_3_ plants at 60 d, showing higher and lower values in roots, respectively (S8 Table).

### δ ^15^N dynamics in plant DNA

δ ^15^N of DNA purified from leaves and roots generally followed a similar pattern as compared to that observed in the source plant materials (Fig 3), resulting from the interaction of plant age and labelling treatment effects (Table 1), although with peculiar and interesting shifts along the observation period. In particular, in the case of leaf DNA, δ ^15^N of ṄH_4_ DNA was consistently higher compared to the other two treatments throughout the first 90 days of observation (S9 Table), exceeding that of ṄH_4_ṄO_3_ DNA by 34%, 49.9%, and 29.8% at 60, 75 and 90 d, respectively, and that of ṄO_3_ DNA by 50.6%, 80.9% and 43%, respectively. Interestingly, such differences were released at 105 d, with δ ^15^N of DNA from all treatments showing converging dynamics up to that point, with a shift in time in comparison to what was observed for the δ ^15^N of the source plant materials (Fig 3). Thereafter, at 105 d and 120 d, δ ^15^N of DNA apparently decreased in all treatments, although such trend was statistically significant limited to ṄH_4_ plants (S9 Table).

Different from leaf DNA, δ ^15^N dynamics in root DNA were much more similar to those observed for the source plant materials, with no significant within-treatment variation between 60 and 90 d, and ṄH_4_ DNA always showing higher levels compared to the other two treatments in the same time frame. In particular, δ ^15^N of ṄH_4_ DNA exceeded that of ṄH_4_ṄO_3_ DNA by 68.6%, 76.5%, and 43.7% at 60, 75 and 90 d, respectively, and that of ṄO_3_ DNA by 196.7%, 213.4% and 99.1%, respectively, corresponding to a larger magnitude of between-treatment variation compared to the source plant materials (Fig 3). From 90 d to 105 d, the δ ^15^N of ṄH_4_ DNA and ṄO_3_ DNA showed abrupt shifts corresponding to significant decrease and increase, respectively, while δ ^15^N did not significantly vary in ṄH_4_ṄO_3_ DNA (Fig 3, S9 Table). Such trends were released at the final observation stage (120 d), as none of the three labelling treatments produced significant variation compared to the preceding stage (105 d) (Fig 3, S9 Table).

Similar to what observed for the total N pools of plant materials, δ ^15^N differences between root and leaf DNA within each labelling treatment at each observation stage were not statistically significant, with the exceptions of ṄO_3_ DNA at 60 d and ṄH_4_ DNA at 60 and 75 d, showing lower and higher values in roots, respectively (S9 Table).

### δ ^15^N NDI dynamics: DNA vs total N pool of the source plant materials

A comparative analysis of δ ^15^N dynamics in DNA samples and in the corresponding source materials can be better clarified by observing δ ^15^N NDI patterns (Fig 3), which significantly changed in relation to the labelling treatment, plant material, age and their interactions (Table 2). In the case of leaves, δ ^15^N NDI values were consistently negative throughout the observation period for ṄH_4_ṄO_3_ and ṄO_3_ plants, indicating that leaf DNA was always depleted in ^15^N compared to the source plant material (S10 Table). Such trend was found in ṄH_4_ leaves only at the final observation stage (120 d), while at the preceding stages δ ^15^N NDI values did not significantly differ from the reference zero value, thus indicating that δ ^15^N of ṄH_4_ DNA did not differ from that of the source plant material (S10 Table). Correspondingly, δ ^15^N NDI dynamics within each treatment did not show significant fluctuations up to the third or fourth observation stage (90 d or 105 d). Then, δ ^15^N NDI significantly decreased, with negative values consistently observed at 120 d in all treatments, indicating that ^15^N depletion in DNA at the final observation stage was independent of the labelling treatment.

**Table 2:** Results of GLM for δ ^15^N NDI.

Effect	DoF	SS	MS	F	*P*
Labelling treatment (L)	2	0.322	0.161	43.443	< 0.0001
Plant material (M)	1	0.091	0.091	24.483	< 0.0001
Plant age (A)	1	0.057	0.057	15.469	0.0001
L × M	2	0.044	0.022	5.922	0.0033
L × A	2	0.182	0.091	24.531	< 0.0001
M × A	1	0.127	0.127	34.362	< 0.0001
L × M × A	2	0.042	0.021	5.614	0.0044
Error	166	0.615	0.004		

GLM includes main and interactive effects of labelling treatment (L, three levels: ṄH_4_ṄO_3_, ṄH_4_ and ṄO_3_), plant material (M, two levels: leaf and root) and age (A, continuous covariate).

δ ^15^N NDI dynamics in plant roots showed substantially the same pattern observed for leaves up to the first 90 d (Fig 3), with the only exception of ṄH_4_ roots showing a significant positive value at 90 d (S10 Table). Differently, at the final observation stages (105 d and 120 d) δ ^15^N NDI dynamics in plant roots showed a significantly different pattern compared to that observed for plant leaves, particularly in the cases of ṄH_4_ṄO_3_ and ṄO_3_ roots (S10 Table). Indeed, ṄH_4_ṄO_3_ roots persistently showed negative δ ^15^N NDI values, but without the decreasing trend observed in ṄH_4_ṄO_3_ leaves (Fig 3). ṄO_3_ roots showed an increasing trend (Fig 3), leading to δ ^15^N NDI values non-significantly different form the reference zero value (S10 Table). ṄH_4_ roots showed substantially the same pattern observed for ṄH_4_ leaves (Fig 3), with a significant decrease from 90 to 120 d corresponding to negative δ ^15^N NDI value at the final stage (S10 Table) indicating ^15^N depletion in DNA.

Considering δ ^15^N NDI values in roots and leaves within each treatment and observation stage, we found the only significant difference at the end of the observation period (120 d), with ṄH_4_ṄO_3_ and ṄO_3_ leaves showing lower values compared to the corresponding root materials (S10 Table), thus indicating that ^15^N depletion in DNA compared to the total N pool was larger in leaves than in roots.

## Discussion

### Effects of N uptake on δ ^15^N dynamics in leaf and root

We found that the isotopic composition of plant roots and leaves largely varied along the vegetative growth period, with early-to-medium dynamics corresponding to ^15^N enrichment and depletion in ṄH_4_ and ṄO_3_ plants, respectively, and with an opposite pattern at later stages. Such trend, more evident in roots compared to leaves, was independent of labelling treatments effects on plant growth and N content and biomass allocation dynamics. The substantially specular dynamics of δ ^15^N in ṄH_4_ vs. ṄO_3_ plants, clearly indicated that uptake fluxes of the two N chemical species were decoupled over time, with plants mostly using NH_4_^+^ up to an age of 90 days and NO_3_^-^ afterwards. This is consistent with the expected outcomes, since the relative abundance and the isotopic composition of different chemical species in the substrate are the most controlling factors of plant δ ^15^N dynamics [30]. In this respect, it is worth noting that our experimental design is novel as compared to previous studies, where a single labelled N species and single harvesting shortly after the labelling treatment were used [4, 30, 41]. Differently, by adopting a factorial combination of different labelled N sources and harvesting plants over a prolonged observation period, we assessed the relative importance of uptake fluxes by comparing enrichment dynamics between ṄH_4_ and ṄO_3_ plants. Additionally, we could evaluate the associated isotopic fractioning by monitoring labelling dynamics in ṄH_4_ṄO_3_. As such, our results clearly confirm that the effects of the N source largely overcome that of isotopic discrimination during N uptake in controlling plant δ ^15^N dynamics.

Consistently, δ ^15^N did not significantly change over time in ṄH_4_ṄO_3_ roots and leaves, as not affected by the uptake dynamics of the two labelled N chemical species. Such finding may be surprising, as at least a slight increase over time in δ ^15^N of ṄH_4_ṄO_3_ plants, and particularly of their roots, would be expected as a result of isotopic discrimination associated to NH_4_^+^ and/or NO_3_^-^ uptake [1], due to a progressive enrichment in ^15^N in the pot solution and hence in plant roots. The magnitude of isotopic discrimination (ε) associated to N uptake was previously estimated both for NO_3_^-^ [13, 41, 42] and NH_4_^+^ [4, 30]. Reported values ranges between 0 (i.e. absence of discrimination) and -12,6 mUr [4, 30] in the case of NH_4_^+^, and between 0 and -9,6 mUr [30, 41, 42] for the uptake of NO_3_^-^. Such variability could be likely related to different experimental conditions considered in those previous studies. Among these, the target species and/or cultivar [7] and plant age [1] may play a major role, as well as N concentration in the substrate, which at low values triggers active uptake transporters [4, 8] and positively affects the magnitude of isotopic discrimination. Therefore, it is not surprising that we could not detect the occurrence of isotopic fractioning associated to N uptake, considering that we started to monitor δ ^15^N values in N-rich plants of 60 d.

### Effects of N assimilation on δ ^15^N dynamics in leaf and root

We expected isotopic enrichment in ṄH_4_ roots compared to leaves and in ṄO_3_ leaves compared to roots at each observation stage, and intermediate values in ṄH_4_ṄO_3_ materials, due to differences in assimilation rates of the two inorganic N chemical species in the two plant organs.

NO_3_^-^ is readily assimilated in the roots after uptake, being firstly reduced to NO_2_^-^ by the Nitrate Reductase enzymatic complex and then to NH_4_^+^ by the Ferrodoxin-Nitrite reductase [8]. The first enzyme discriminates the heavy isotopic form [1, 9], with a generally accepted fractionation value of –16 mUr [11, 14]. Then, the NO_3_^-^ available for assimilation in leaves, after xylematic transport, is enriched in ^15^N compared to that assimilated at root level [1]. Our results confirmed the expected pattern only for the first observation stage (60 d). This apparently contrasts with the enhanced content of assimilation enzymes at root level [43], which is expected to result in progressive enrichment of unassimilated NO_3_^-^ that is then transported to the leaves. However, our results can be easily explained considering the remarkable N availability in the pot solution and the consequent low NO_3_^-^ assimilation flux in the roots. In such conditions, biomass and N allocation were extremely unbalanced between roots and leaves. For instance, at a plant age of 75 d, mean N content values in ṄO_3_ leaves and roots were 111.8 mg and 3.9 mg, respectively, while the same values at 90 d were 177.4 mg and 12.3 mg. This corresponded to 65.6 mg N allocated to the leaves, while the net N allocation to the roots in the same time frame was one order of magnitude lower, equal to only 8.4 mg. Therefore, ^15^N enrichment in the leaves due to fractioning effects of NO_3_^-^ assimilation in the roots [1] was likely lower than expected, as related to the low NO_3_^-^ assimilation flux in the roots. In addition, a ^15^N enrichment in roots due to the backflow of nitrate to roots via the phloem, as recently suggested by a modelling work by [14], cannot be excluded.

In the case of NH_4_^+^ assimilation, N is firstly incorporated into organic molecules such as Glutamine and Glutamate in the roots [8, 44] through a three-stages assimilation process mediated by the GS-GOGAT enzymatic complex. Since the first stage of such process, mediated by the Glutamine Synthetase enzyme, discriminates the heavy N isotopic form [e.g. 12, 45], the organic products of the NH_4_^+^ assimilation transported to the leaves are depleted in ^15^N. Therefore, δ ^15^N in ṄH_4_ roots is expected to be higher than in ṄH_4_ leaves. Our results for ṄH_4_ plants were consistent with such expectation only at the first observation stage (60 d). Interestingly, at later stages, we found large within-group variability, particularly at 90 d. At this stage, δ ^15^N also did not differ among ṄH_4_ and ṄO_3_ materials, indicating that plant materials had acquired the same amount of NH_4_^+^ and NO_3_^-^, irrespective of the labelling treatment. Since at this stage slight variations in the uptake rates of either N source produces large δ ^15^N variations, the high within-group variability of δ ^15^N values, which prevented from detecting significant root vs. leaf differences, could be ascribed to a certain asynchrony in labelling dynamics among replicates within each treatment. Finally, a possible role of ammonia volatilization, at least for some replicates, and its effect on δ ^15^N dynamics cannot be excluded [12]. It is well known that ammonia volatilization rates increase with temperature and with leaf N content, particularly during senescence [46, 47]. In our experiment, mild temperatures and absence of senescence processes likely contributed to limit ammonia volatilization. On the other hand, the remarkable percent N content in plant leaves up to the end of the observation period, might have enhanced N loss by volatilization.

### ^15^N depletion dynamics in plant DNA

Following Gauthier et al. [20], we hypothesized that isotopic fractioning along the purine and/or pyrimidine biosynthesis pathways leads to a depletion of ^15^N in plant DNA, hence expecting lower δ ^15^N values in leaf and root DNA samples compared to those of the source plant materials.

Dynamics of δ ^15^N values in DNA samples, decreasing and increasing in ṄO_3_ and ṄH_4_ treatments, respectively, were substantially consistent to those of the source materials up to 105 d. Then, at the final observation period, δ ^15^N dynamics in all DNA samples were completely different from that of the source materials, with a consistent decrease in most cases. Dynamics of δ ^15^N NDI provided a clue to explain such pattern, consistently showing negative values for ṄH_4_ṄO_3_ and ṄO_3_ (i.e. ^15^N depletion in DNA samples compared to the total N pool of the source plant materials) for both leaves and roots. For such treatments, our findings fully support the occurrence of isotopic fractioning along the purine and/or pyrimidine biosynthesis pathways [20]. On the other hand, δ ^15^N NDI dynamics observed in the case of ṄH_4_ leaves and roots, showed values not significantly different from zero (i.e. equal δ ^15^N in DNA samples and the source plant materials up to 105 d), and even a positive value for roots at 90d. Such result may be explained considering the interplay of isotopic fractioning during DNA biosynthesis, N uptake and assimilation in the three labelling treatments. Up to 90 d, the negative δ ^15^N NDI values of ṄH_4_ṄO_3_ plants, as not affected by the labelled N species, relied only on ^15^N depletion in DNA. Differently, δ ^15^N NDI values were significantly different between ṄO_3_ and ṄH_4_ plants, for both leaves and roots. This indicates that the progressive isotopic enrichment and depletion in ṄO_3_ and ṄH_4_ leaves and roots, respectively, were exacerbated in DNA samples compared to the source materials. Moreover, the increase and decrease of δ ^15^N values in ṄO_3_ and ṄH_4_ DNA samples, respectively, were delayed compared to the corresponding plant materials. This may be attributed to a temporal decoupling of N incorporation in purine and pyrimidine precursors with respect to nucleotide assemblage into DNA molecules, with ^15^N signature of DNA samples at a given stage reflecting that of the source material at previous stages. Accordingly, in the case of ṄH_4_ DNA samples, the effect of isotopic fractioning in purine and pyrimidine biosynthesis [20] might have been masked by labelling dynamics of the source materials.

After 90 d, δ ^15^N NDI values were consistently negative, and mostly decreasing, for leaves of all treatments, confirming the occurrence of ^15^N fractioning during leaf DNA biosynthesis [20]. The results for root materials was less straightforward, for different possible reasons. First, root growth rate between 105 and 120 d was one order of magnitude smaller compared to leaves (mean and standard deviation of all treatments was *k*_*B*_ = 0.036 ± 0.011 g_DW_ d^-1^ and *k*_*B*_ = 0.353 ± 0.020 g_DW_ d^-1^ for roots and leaves, respectively). In these conditions, DNA biosynthesis rate was higher in leaves, compared to roots and to the preceding stages, hence magnifying the effect of isotopic fractioning associated to purine and pyrimidine biosynthesis. Second, in the same observation period (between 105 and 120 d) mean daily increases of N mass in leaves (*k*_*N*_ = 0.58 ± 0.1 mg_N_ d^-1^) and roots (*k*_*N*_ = 0.18 ± 0.03 mg_N_ d^-1^) showed more similar magnitude as compared to the corresponding *k*_*B*_ values. Hence, at this stage, the utilization efficiency [48] of newly up taken N (i.e. *k*_*B*_ / *k*_*N*_) in the leaves (i.e. 0.222 g_DW_ mg_N_^-1^) was larger compared to the roots (0.077 g_DW_ mg_N_^-1^) and to the preceding stages (i.e. 0.012, 0.013, 0.024 and 0.031 g_DW_ mg_N_^-1^ at 60, 75, 90 and 105 d, respectively), with DNA bases biosynthesis likely relying more on this N pool rather than that previously taken up but not used and stored in reserve pools such as vegetative proteins [32]. Finally, we cannot exclude a possible and decisive role of N translocation from leaves to roots [44], which however should have played the major role at the initial stages of observation, when percent N content in leaves was far higher than physiological values commonly reported [e.g. 32].

## Conclusion

In this study we confirmed previous evidence on the effect of the labelled chemical species on leaf and root δ ^15^N dynamics. Under the tested conditions, higher uptake rate of NH_4_^+^ and its limiting effect on the uptake of NO_3_^-^ were the main causal factors of the observed outcomes, with ṄH_4_, ṄO_3_ and ṄH_4_ṄO_3_ plants initially showing higher, lower and intermediate δ ^15^N values, respectively, then progressing towards the opposite trend when NH_4_^+^ depletion from the nutrient solution corresponded to increasing NO_3_^-^ uptake rate.

Although it is well known that isotopic fractionation during inorganic N uptake, associated to ^15^N enrichment of the N pool residual in the substrate solution, results in progressive isotopic enrichment of plant tissues, our study did not provide conclusive results, even in the case of ṄH_4_ṄO_3_ plants, unaffected by uptake rates of the two chemical species. However, possibly unsuitable experimental conditions, in terms of excessive N availability, might have hampered active inorganic N uptake mechanisms, decisively affecting our observations.

Evidence from previous studies on leaf vs. root isotopic enrichment due to enzymatic fractionation during inorganic N assimilation were only partially confirmed, limited to ṄO_3_ plants at the early observation stages. At later stages and for ṄH_4_^+^ plants, the predominant effects of NH_4_^+^ and NO_3_^-^ uptake rates in the tested conditions, as well as the reduced root development and the extremely high leaf N content, with the associated possible confounding effects of nitrate phloematic backflow and ammonia volatilization, likely masked the expected outcome.

Considering the hypothesis of ^15^N depletion in DNA compared to the source plant materials, possibly due to enzymatic discrimination during purine biosynthesis, our findings provide confirmatory evidence. However, we did not provide a direct evidence of δ ^15^N variation between molecular products such as nuclei acids and their precursors according to known biochemical pathways. Indeed, addressing such issue would require more detailed characterization of the involved N molecular pools and additional experiments to accurately estimate the fractionation coefficient of each enzymatic step during DNA biosynthesis. However, as an added value of our original experimental design, we were able for the first time to specifically report about the dynamics of specific plant molecular pools, such as leaf and root DNA, over a long observation period.

## Supporting information

S1 Fig Shoot: Root ratio changes of in *B*. *napus* plants over the observation period, across the labelling treatments. Different letters above bars indicate significant pair-wise labelling-dependent differences at equal plant age (Tuckey’s post-hoc test after two-ways ANOVA, S6 and S7 Tables).
(TIF)

S1 Table Physical-chemical features of the quartz sand substrate used for potting. (PDF)

S2 Table Results of GLM for *B*. *napus* biomass and N percent content. (PDF)

S3 Table Result of Tuckey’s post-hoc HSD testing for the interactive effect of plant age and labelling treatments (ṄH_4_ṄO_3_, ṄH_4_, ṄO_3_) on dry biomass of *B*. *napus* leaves, stems and roots. Data refer to mean ± standard deviation of dry weight (g) of 6 plants for each treatment combination. Different letters indicate significantly different groups within each plant material (P < 0.05).
(PDF)

S4 Table Result of Tuckey’s post-hoc HSD testing for the interactive effect of plant age and labelling treatments (ṄH_4_ṄO_3_, ṄH_4_, ṄO_3_) on percent N content in *B*. *napus* leaves, stems and roots. Data refer to mean ± standard deviation of N content (%) of 6 plants for each treatment combination. Different letters indicate significantly different groups within each plant material (P < 0.05).
(PDF)

S5 Table Leaf, stem and root biomass and percent N content in the unlabelled control plants at the five observation stages. (PDF)

S6 Table Result of two-ways ANOVA testing for main and interactive effects of plant age and labelling treatment (ṄH_4_ṄO_3_, ṄH_4_, ṄO_3_) on the shoot: Root ratio of *B*. *napus* plants. (PDF)

S7 Table Result of Tuckey’s post-hoc testing for the interactive effect of plant age and labelling treatment (ṄH_4_ṄO_3_, ṄH_4_, ṄO_3_) on the shoot: Root ratio of *B*. *napus* plants. (PDF)

S8 Table Result of Tuckey’s post-hoc HSD testing for the interactive effect of plant age and labelling treatments (ṄH_4_ṄO_3_, ṄH_4_, ṄO_3_) on N isotopic composition of *B*. *napus*. Data refer to mean ± standard deviation of 6 plants for each treatment combination. Different letters indicate significantly different groups within each plant material (P < 0.05). Significantly different values between leaf and root within each combination of labelling treatment and plant age are indicated in bold.
(PDF)

S9 Table Result of Tuckey’s post-hoc HSD testing for the interactive effect of plant age and labelling treatments (ṄH_4_ṄO_3_, ṄH_4_, ṄO_3_) on δ ^15^N of *B*. *napus* leaf and root DNA. Data refer to δ ^15^N mean ± standard deviation of 6 plants for each treatment combination. Different letters indicate significantly different groups within each plant material (P < 0.05). Significantly different values between leaf and root within each combination of labelling treatment and plant age are indicated in bold (*: DNA purified from root materials was pooled in order to provide the minimum sample amount for IRMS analysis).
(PDF)

S10 Table Result of Tuckey’s post-hoc HSD testing for the interactive effect of plant age and labelling treatments (ṄH_4_ṄO_3_, ṄH_4_, ṄO_3_) on δ ^15^N NDI. δ ^15^N NDI indicates differences of isotopic composition between leaf or root DNA and the total N pool of the source plant material. Data refer to δ ^15^N NDI mean ± standard deviation of 6 plants for each treatment combination. Different letters indicate significantly different groups within each plant material (P < 0.05). Mean values significantly different from zero, as assessed by one sample t-tests at P < 0.01, are marked with an asterisk (*). Significantly different values between leaf and root within each combination of labelling treatment and plant age are indicated in bold.
(PDF)
