# Supplementary figures and images for: N uptake, assimilation and isotopic fractioning control δ 15N dynamics in plant DNA: A heavy labelling experiment on Brassica napus L
Source: PLoS One. 2021 Mar 11;16(3):e0247842. doi: 10.1371/journal.pone.0247842 (PMC7951814; doi:10.1371/journal.pone.0247842)

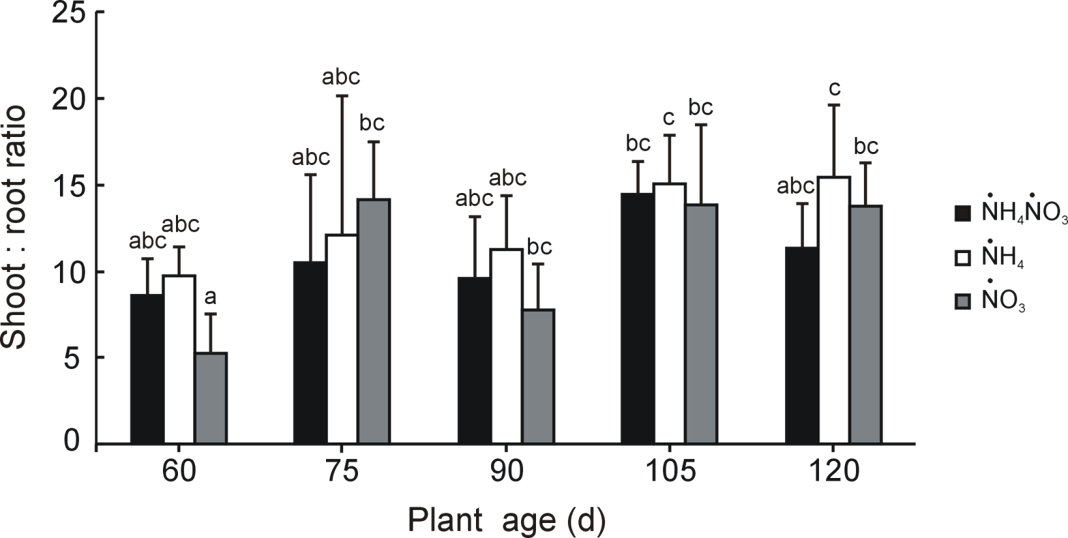

Supplement: S1 Fig — Different letters above bars indicate significant pair-wise labelling-dependent differences at equal plant age (Tuckey’s post-hoc test after two-ways ANOVA, S6 and S7 Tables). (TIF) [file pone.0247842.s001.tif]
