# Supplementary material for: N uptake, assimilation and isotopic fractioning control δ 15N dynamics in plant DNA: A heavy labelling experiment on Brassica napus L
Source: PLoS One. 2021 Mar 11;16(3):e0247842. doi: 10.1371/journal.pone.0247842 (PMC7951814; doi:10.1371/journal.pone.0247842)
Supplement: S1 Table — (PDF) [file pone.0247842.s002.pdf]

S1 Table. Physical-chemical features of the quartz sand substrate used for potting.

Characteristic	Value
Particle size	≤ 1 mm
Density	2.65 g/cm^3
Cl content	< 0.01 %
Humus content	Absent
Total S content	< 1 %
$CO_3^=$	10.45 %
Total N	< 0.01 %
Light contaminants	Absent
