# Supplementary material for: N uptake, assimilation and isotopic fractioning control δ 15N dynamics in plant DNA: A heavy labelling experiment on Brassica napus L
Source: PLoS One. 2021 Mar 11;16(3):e0247842. doi: 10.1371/journal.pone.0247842 (PMC7951814; doi:10.1371/journal.pone.0247842)
Supplement: S2 Table — (PDF) [file pone.0247842.s003.pdf]

S2 Table. Results of GLM for *B. napus* biomass and N percent content.

Effect	DoF	SS	MS	F	*p*
Biomass					
Labelling treatment (L)	2	0.462	0.231	0.244	0.7841
Plant material (M)	2	149.794	74.897	78.914	< 0.0001
Plant age (A)	1	627.602	627.602	661.262	< 0.0001
L × M	4	1.762	0.441	0.464	0.7620
L × A	2	0.254	0.127	0.134	0.8747
M × A	2	376.688	188.344	198.445	< 0.0001
L × M × A	4	1.549	0.387	0.408	0.8027
Error	223	211.649	0.949		
N content (%)					
Labelling treatment (L)	2	0.247	0.124	0.154	0.8572
Plant material (M)	2	239.095	119.547	148.999	< 0.0001
Plant age (A)	1	645.382	645.382	804.378	< 0.0001
L × M	4	0.174	0.044	0.054	0.9945
L × A	2	0.284	0.142	0.177	0.8378
M × A	2	110.420	55.210	68.812	< 0.0001
L × M × A	4	0.187	0.047	0.058	0.9937
Error	234	187.747	0.802		

GLMs include main and interactive effects of labelling treatment (L, three levels: $\dot{N}H_4\dot{N}O_3$, $\dot{N}H_4$, $\dot{N}O_3$), plant material (M, three levels: leaf, stem and root) and age (A, continuous covariate).
