# Supplementary material for: N uptake, assimilation and isotopic fractioning control δ 15N dynamics in plant DNA: A heavy labelling experiment on Brassica napus L
Source: PLoS One. 2021 Mar 11;16(3):e0247842. doi: 10.1371/journal.pone.0247842 (PMC7951814; doi:10.1371/journal.pone.0247842)
Supplement: S3 Table — Data refer to mean ± standard deviation of dry weight (g) of 6 plants for each treatment combination. Different letters indicate significantly different groups within each plant material (P < 0.05). (PDF) [file pone.0247842.s004.pdf]

S3 Table. Result of Tuckey's post-hoc HSD testing for the interactive effect of plant age and labelling treatments ($\dot{N}H_4\dot{N}O_3$, $\dot{N}H_4$, $\dot{N}O_3$) on dry biomass of *B. napus* leaves, stems and roots.

Plant material	Plant age (days)	Dry biomass (g)		
		$\dot{N}H_4\dot{N}O_3$	$\dot{N}H_4$	$\dot{N}O_3$
Leaf	60	1.10 ± 0.20 *ab*	1.20 ± 0.33 *abc*	1.00 ± 0.05 *abc*
	75	1.78 ± 0.32 *abc*	1.61 ± 0.33 *abc*	1.27 ± 0.09 *abc*
	90	3.10 ± 1.34 *bcde*	3.21 ± 1.54 *cdef*	3.01 ± 1.16 *bcd*
	105	5.27 ± 0.97 *f*	5.41 ± 1.48 *ef*	5.22 ± 1.62 *ef*
	120	10.71 ± 3.68 *g*	10.37 ± 2.88 *g*	10.73 ± 0.85 *g*
Stem	60	0.28 ± 0.05 *a*	0.28 ± 0.07 *a*	0.22 ± 0.02 *a*
	75	0.49 ± 0.09 *a*	0.48 ± 0.11 *a*	0.44 ± 0.06 *a*
	90	1.38 ± 0.59 *a*	1.28 ± 0.78 *a*	1.24 ± 0.53 *a*
	105	2.11 ± 0.41 *ab*	1.97 ± 0.49 *ab*	1.94 ± 0.37 *ab*
	120	3.84 ± 1.21 *bc*	3.98 ± 1.48 *c*	3.66 ± 0.22 *bc*
Root	60	0.17 ± 0.07 *a*	0.16 ± 0.05 *a*	0.23 ± 0.12 *a*
	75	0.25 ± 0.11 *a*	0.25 ± 0.18 *a*	0.15 ± 0.04 *a*
	90	0.43 ± 0.21 *a*	0.44 ± 0.27 *a*	0.49 ± 0.22 *a*
	105	0.51 ± 0.09 *a*	0.56 ± 0.17 *a*	0.51 ± 0.16 *a*
	120	1.24 ± 0.52 *a*	0.96 ± 0.29 *a*	0.98 ± 0.36 *a*

Data refer to mean ± standard deviation of dry weight (g) of 6 plants for each treatment combination. Different letters indicate significantly different groups within each plant material ($P < 0.05$).
