# Supplementary material for: N uptake, assimilation and isotopic fractioning control δ 15N dynamics in plant DNA: A heavy labelling experiment on Brassica napus L
Source: PLoS One. 2021 Mar 11;16(3):e0247842. doi: 10.1371/journal.pone.0247842 (PMC7951814; doi:10.1371/journal.pone.0247842)
Supplement: S4 Table — Data refer to mean ± standard deviation of N content (%) of 6 plants for each treatment combination. Different letters indicate significantly different groups within each plant material (P < 0.05). (PDF) [file pone.0247842.s005.pdf]

S4 Table. Result of Tuckey's post-hoc HSD testing for the interactive effect of plant age and labelling treatments ($\dot{N}H_4\dot{N}O_3$, $\dot{N}H_4$, $\dot{N}O_3$) on percent N content in *B. napus* leaves, stems and roots.

Plant material	Plant age (days)	N content (%)		
		$\dot{N}H_4\dot{N}O_3$	$\dot{N}H_4$	$\dot{N}O_3$
Leaf	60	8.36 ± 0.44 *c*	8.47 ± 0.4 *c*	8.32 ± 0.53 *c*
	75	8.09 ± 0.43 *c*	8.15 ± 0.86 *c*	8.43 ± 0.14 *c*
	90	6.12 ± 1.51 *b*	6.11 ± 1.32	6.41 ± 1.14 *bc*
	105	4.85 ± 0.46 *b*	4.98 ± 1.20 *b*	5.06 ± 0.70 *b*
	120	2.63 ± 1.11 *a*	2.79 ± 0.90 *a*	2.70 ± 0.26 *a*
Stem	60	6.20 ± 1.06 *b*	6.29 ± 0.76 *b*	6.14 ± 0.68 *ab*
	75	6.55 ± 0.24 *ab*	6.44 ± 0.54 *ab*	6.66 ± 0.62 *ab*
	90	4.63 ± 1.57 *ab*	4.75 ± 1.18 *ab*	4.61 ± 1.07 *ab*
	105	2.78 ± 0.59 *ab*	2.71 ± 1.47 *ab*	2.45 ± 0.13 *ab*
	120	0.81 ± 0.46 *a*	0.86 ± 0.59 *a*	0.85 ± 0.14 *a*
Root	60	3.81 ± 1.20 *cd*	3.80 ± 0.71 *bcd*	3.46 ± 1.69 *cd*
	75	2.87 ± 0.60 *d*	2.95 ± 0.79 *bcd*	3.02 ± 0.63 *d*
	90	2.37 ± 0.68 *bc*	2.71 ± 0.83 *bcd*	2.64 ± 0.53 *bc*
	105	2.08 ± 0.22 *ab*	2.20 ± 0.38 *a*	1.96 ± 0.87 *ab*
	120	1.57 ± 0.43 *a*	1.68 ± 0.43 *a*	1.84 ± 0.19 *a*

Data refer to mean ± standard deviation of N content (%) of 6 plants for each treatment combination. Different letters indicate significantly different groups within each plant material ($P < 0.05$).
