# Supplementary material for: N uptake, assimilation and isotopic fractioning control δ 15N dynamics in plant DNA: A heavy labelling experiment on Brassica napus L
Source: PLoS One. 2021 Mar 11;16(3):e0247842. doi: 10.1371/journal.pone.0247842 (PMC7951814; doi:10.1371/journal.pone.0247842)
Supplement: S5 Table — (PDF) [file pone.0247842.s006.pdf]

S5 Table. Leaf, stem and root biomass and percent N content in the unlabelled control plants at the five observation stages.

Plant material	Plant age (days)	Biomass (g, DW)	N content (%)
Leaf	60	1.21 ± 0.43	7.70 ± 1.24
	75	2.20 ± 1.04	8.22 ± 0.12
	90	2.91 ± 2.26	7.02 ± 0.19
	105	4.45 ± 3.45	6.53 ± 1.67
	120	16.40 ± 1.46	1.95 ± 0.02
Stem	60	0.25 ± 0.08	8.52 ± 1.15
	75	0.62 ± 0.35	7.81 ± 0.71
	90	0.94 ± 0.59	5.23 ± 0.30
	105	1.45 ± 1.20	3.69 ± 1.53
	120	5.19 ± 0.34	0.65 ± 0.05
Root	60	0.14 ± 0.09	2.71 ± 0.52
	75	0.66 ± 0.50	1.72 ± 1.46
	90	0.64 ± 0.59	0.98 ± 0.22
	105	0.48 ± 0.24	1.85 ± 0.06
	120	2.06 ± 0.61	1.51 ± 0.01

Data refer to mean ± standard deviation of 2 control plants for each observation stage.
