# Supplementary material for: N uptake, assimilation and isotopic fractioning control δ 15N dynamics in plant DNA: A heavy labelling experiment on Brassica napus L
Source: PLoS One. 2021 Mar 11;16(3):e0247842. doi: 10.1371/journal.pone.0247842 (PMC7951814; doi:10.1371/journal.pone.0247842)
Supplement: S6 Table — (PDF) [file pone.0247842.s007.pdf]

S6 Table. Result of two-ways ANOVA testing for main and interactive effects of plant age and labelling treatment ($\dot{N}H_4\dot{N}O_3$, $\dot{N}H_4$, $\dot{N}O_3$) on the shoot : root ratio of *B. napus* plants.

Effect	DoF	SS	MS	F	*p*
Shoot/root (S/R)					
Plant age (A)	4	547.94	136.99	10.9350	< 0.0001
Labelling treatment (L)	2	63.61	31.80	2.5388	0.0857
A x L	8	132.66	16.58	1.3237	0.2450
Error	75	939.54	12.53		
