# Supplementary material for: N uptake, assimilation and isotopic fractioning control δ 15N dynamics in plant DNA: A heavy labelling experiment on Brassica napus L
Source: PLoS One. 2021 Mar 11;16(3):e0247842. doi: 10.1371/journal.pone.0247842 (PMC7951814; doi:10.1371/journal.pone.0247842)
Supplement: S7 Table — (PDF) [file pone.0247842.s008.pdf]

S7 Table. Result of Tuckey's post-hoc testing for the interactive effect of plant age and labelling treatment ($\dot{N}H_4\dot{N}O_3$, $\dot{N}H_4$, $\dot{N}O_3$) on the shoot : root ratio of *B. napus* plants.

Plant age (days)	Labelling treatments		
	$\dot{N}H_4\dot{N}O_3$	$\dot{N}H_4$	$\dot{N}O_3$
60	8.6 ± 2.1 *abc*	9.7 ± 1.6 *abc*	5.3 ± 2.2 *a*
75	10.5 ± 4.8 *abc*	12.1 ± 7.6 *abc*	14.2 ± 3.1 *bc*
90	9.6 ± 3.4 *abc*	11.3 ± 3 *abc*	7.8 ± 2.5 *ab*
105	14.5 ± 1.8 *bc*	15 ± 2.7 *c*	13.8 ± 4.4 *bc*
120	11.3 ± 2.4 *abc*	15.4 ± 4 *c*	13.7 ± 2.4 *bc*
