# Supplementary material for: N uptake, assimilation and isotopic fractioning control δ 15N dynamics in plant DNA: A heavy labelling experiment on Brassica napus L
Source: PLoS One. 2021 Mar 11;16(3):e0247842. doi: 10.1371/journal.pone.0247842 (PMC7951814; doi:10.1371/journal.pone.0247842)
Supplement: S8 Table — Data refer to mean ± standard deviation of 6 plants for each treatment combination. Different letters indicate significantly different groups within each plant material (P < 0.05). Significantly different values between leaf and root within each combination of labelling treatment and plant age are indicated in bold. (PDF) [file pone.0247842.s009.pdf]

S8 Table. Result of Tuckey's post-hoc HSD testing for the interactive effect of plant age and labelling treatments ($\dot{N}H_4\dot{N}O_3$, $\dot{N}H_4$, $\dot{N}O_3$) on N isotopic composition of *B. napus*.

Plant material	Plant age (days)	N isotopic composition of plant materials ($\delta^{15}N_{Air\text{-}N2}$, mUr or ‰)		
		$\dot{N}H_4\dot{N}O_3$	$\dot{N}H_4$	$\dot{N}O_3$
Leaf	60	2213.4 ± 61.5 *abc*	**2587.6 ± 177.5 *cde***	**2291.9 ± 262.9 *abcd***
	75	2186.8 ± 35.5 *abc*	2801.8 ± 181.2 *de*	2028.9 ± 169.0 *ab*
	90	2233.0 ± 25.6 *abc*	2236.4 ± 367.1 *abc*	2380.5 ± 400.9 *bcde*
	105	2242.3 ± 32.6 *abc*	2002.7 ± 183.3 *ab*	2681.1 ± 259.8 *cde*
	120	2175.5 ± 41.2 *abc*	1764.7 ± 360.3 *a*	2851.3 ± 201.2 *e*
Root	60	2193.3 ± 69.5 *bcde*	**3267.4 ± 139.8 *f***	**1730.4 ± 255.9 *ab***
	75	2098.1 ± 54.0 *abcde*	3170.8 ± 427.3 *f*	1634.4 ± 161.1 *a*
	90	2174.6 ± 24.1 *abcde*	2549.4 ± 437.9 *e*	2143.6 ± 455.0 *abcde*
	105	2131.8 ± 36.7 *abcde*	1957.5 ± 212.7 *abcd*	2315.2 ± 384.4 *cde*
	120	2054.7 ± 23.8 *abcde*	1824.9 ± 501.4 *abc*	2460.7 ± 212.8 *de*

Data refer to mean ± standard deviation of 6 plants for each treatment combination. Different letters indicate significantly different groups within each plant material ($P < 0.05$). Significantly different values between leaf and root within each combination of labelling treatment and plant age are indicated in bold.
