# Supplementary material for: N uptake, assimilation and isotopic fractioning control δ 15N dynamics in plant DNA: A heavy labelling experiment on Brassica napus L
Source: PLoS One. 2021 Mar 11;16(3):e0247842. doi: 10.1371/journal.pone.0247842 (PMC7951814; doi:10.1371/journal.pone.0247842)
Supplement: S9 Table — Data refer to δ 15N mean ± standard deviation of 6 plants for each treatment combination. Different letters indicate significantly different groups within each plant material (P < 0.05). Significantly different values between leaf and root within each combination of labelling treatment and plant age are indicated in bold (*: DNA purified from root materials was pooled in order to provide the minimum sample amount for IRMS analysis). (PDF) [file pone.0247842.s010.pdf]

S9 Table. Result of Tuckey's post-hoc HSD testing for the interactive effect of plant age and labelling treatments ($\dot{N}H_4\dot{N}O_3$, $\dot{N}H_4$, $\dot{N}O_3$) on δ ^{15}N of *B. napus* leaf and root DNA.

Plant material	Plant age (days)	N isotopic composition of DNA ($\delta^{15}N_{Air\text{-}N2}$, mUr or ‰)		
		$\dot{N}H_4\dot{N}O_3$	$\dot{N}H_4$	$\dot{N}O_3$
Leaf	60	1946.4 ± 45.1 *bc*	**2609.1 ± 140.9 *ef***	**1732.8 ± 244.0 *abc***
	75	1974.7 ± 58.8 *bcd*	**2960.3 ± 255.3 *fg***	1636.3 ± 66.1 *abc*
	90	1949.6 ± 132.0 *bc*	2530.0 ± 369.6 *def*	1768.9 ± 290.2 *abc*
	105	1711.4 ± 208.5 *abc*	1908.6 ± 264.1 *bc*	2184.7 ± 253.6 *cde*
	120	1415.3 ± 365.2 *ab*	1238.7 ± 262.7 *a*	1926.7 ± 204.7 *bc*
Root	60	1954.6 ± 80.6 *bcde*	**3294.9 ± 98.8 *gh***	**1110.5 ± 71.4 *a***
	75	2033.2 ± 46.0 *bcde*	**3587.9 ± 381.5 *h***	1144.7 ± 0.0*, *a*
	90	2065.9 ± 47.4 *cde*	2968.8 ± 615.3 *fg*	1491.4 ± 650.7 *ab*
	105	1924.8 ± 44.0 *bcde*	1719.5 ± 131.9 *bcd*	2125.7 ± 422.3 *de*
	120	1789.2 ± 64.0 *bcde*	1537.3 ± 307.9 *abc*	2353.1 ± 317.1 *e*

Data refer to δ ^{15}N mean ± standard deviation of 6 plants for each treatment combination. Different letters indicate significantly different groups within each plant material ($P < 0.05$). Significantly different values between leaf and root within each combination of labelling treatment and plant age are indicated in bold (*: DNA purified from root materials was pooled in order to provide the minimum sample amount for IRMS analysis).
