# Supplementary material for: N uptake, assimilation and isotopic fractioning control δ 15N dynamics in plant DNA: A heavy labelling experiment on Brassica napus L
Source: PLoS One. 2021 Mar 11;16(3):e0247842. doi: 10.1371/journal.pone.0247842 (PMC7951814; doi:10.1371/journal.pone.0247842)
Supplement: S10 Table — δ 15N NDI indicates differences of isotopic composition between leaf or root DNA and the total N pool of the source plant material. Data refer to δ 15N NDI mean ± standard deviation of 6 plants for each treatment combination. Different letters indicate significantly different groups within each plant material (P < 0.05). Mean values significantly different from zero, as assessed by one sample t-tests at P < 0.01, are marked with an asterisk (*). Significantly different values between leaf and root within each combination of labelling treatment and plant age are indicated in bold. (PDF) [file pone.0247842.s011.pdf]

S10 Table. Result of Tuckey's post-hoc HSD testing for the interactive effect of plant age and labelling treatments ($\dot{N}H_4\dot{N}O_3$, $\dot{N}H_4$, $\dot{N}O_3$) on δ ^{15}N NDI.

Plant material	Plant age (days)	δ ^{15}N Normalized Difference Index (NDI)		
		$\dot{N}H_4\dot{N}O_3$	$\dot{N}H_4$	$\dot{N}O_3$
Leaf	60	-0.0641 ± 0.020*, *cde*	0.0045 ± 0.022 *ef*	-0.1404 ± 0.035*, *abc*
	75	-0.0511 ± 0.016*, *cde*	0.0253 ± 0.016 *ef*	-0.1059 ± 0.041*, *bcd*
	90	-0.0686 ± 0.038*, *cde*	0.0627 ± 0.041 *f*	-0.1471 ± 0.017*, *abc*
	105	-0.1368 ± 0.067*, *abc*	-0.0264 ± 0.027 *def*	-0.1030 ± 0.023*, *bcd*
	120	**-0.2226 ± 0.126***, *a*	-0.1747 ± 0.083*, *ab*	**-0.2195 ± 0.046***, *a*
Root	60	-0.0711 ± 0.013*, *c*	0.0044 ± 0.030 *cd*	-0.2151 ± 0.044*, *a*
	75	-0.0216 ± 0.012*, *cd*	0.0635 ± 0.056 *d*	-0.1739 ± 0.049*, *ab*
	90	-0.0257 ± 0.010*, *cd*	0.0729 ± 0.029*, *d*	-0.2033 ± 0.111*, *a*
	105	-0.0511 ± 0.012*, *c*	-0.0632 ± 0.047 *c*	-0.0462 ± 0.036 *c*
	120	**-0.0693 ± 0.018***, *c*	-0.0798 ± 0.033*, *bc*	**-0.0248 ± 0.044** *cd*

δ ^{15}N NDI indicates differences of isotopic composition between leaf or root DNA and the total N pool of the source plant material. Data refer to δ ^{15}N NDI mean ± standard deviation of 6 plants for each treatment combination. Different letters indicate significantly different groups within each plant material ($P < 0.05$). Mean values significantly different from zero, as assessed by one sample t-tests at $P < 0.01$, are marked with an asterisk (*). Significantly different values between leaf and root within each combination of labelling treatment and plant age are indicated in bold.
